# Recent Progress in Emerging Two-Dimensional Transition Metal Carbides

**DOI:** 10.1007/s40820-021-00710-7

**Published:** 2021-08-20

**Authors:** Tianchen Qin, Zegao Wang, Yuqing Wang, Flemming Besenbacher, Michal Otyepka, Mingdong Dong

**Affiliations:** 1 College of Materials Science and Engineering, Sichuan University, Chengdu, 610065 People’s Republic of China; 2 Interdisciplinary Nanoscience Center, Aarhus University, 8000 Aarhus, Denmark; 3 Regional Centre of Advanced Technologies and Materials, Department of Physical Chemistry, Faculty of Science, Palacký University, 77146 Olomouc, Czech Republic

**Keywords:** Two-dimensional transition metal carbides, Phase diagram, Superconductivity, Energy conversation and storage, Large-scale synthesis

## Abstract

As a new member in two-dimensional materials family, transition metal carbides (TMCs) have many excellent properties, such as chemical stability, in-plane anisotropy, high conductivity and flexibility, and remarkable energy conversation efficiency, which predispose them for promising applications as transparent electrode, flexible electronics, broadband photodetectors and battery electrodes. However, up to now, their device applications are in the early stage, especially because their controllable synthesis is still a great challenge. This review systematically summarized the state-of-the-art research in this rapidly developing field with particular focus on structure, property, synthesis and applicability of TMCs. Finally, the current challenges and future perspectives are outlined for the application of 2D TMCs.

## Highlights

- The phase diagram of transition metal carbides (TMCs) is discussed.
- The physical and chemical property of TMCs is systematically summarized.
- The potential application and controllable synthesis of TMCs is discussed.
- A summary is provided to afford the principle to further investigation.

## Graphical abstract

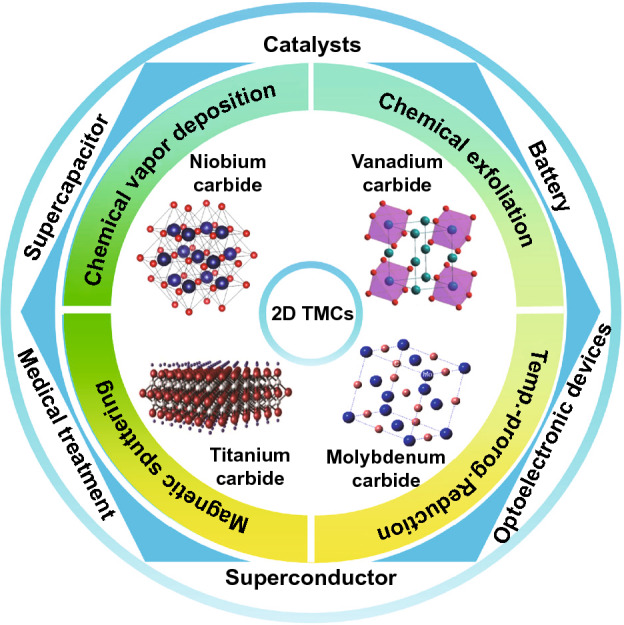

## Introduction

Ultra-thin two-dimensional (2D) nanomaterials, as a new kind of nanomaterials, have been widely concerned for a long time. This is because they have a special planar structure, where the horizontal size is beyond sub-micrometer; however, the vertical size limits in nanometer or even atomic level. As early as 200 years ago, it was found that there were some special layered minerals in nature, which can be peeled off to obtain a new lamellar structure [1]. In 2004, K. S. Novoselov, A. K. Geim and other collaborators reported the fabrication of graphite single layer named graphene by using a special scotch micromechanical stripping [2]. The graphene shows many unique properties, namely extraordinary carrier mobility of up to 200,000 cm^2^ (V s)^−1^, the large specific surface area of 2630 m^2^ g^−1^, the transmittance of 97.7%, the Young’s modulus of 1 TPa and the thermal conductivity of 3000 W (m K) ^−1^ [3, 4]. Ultra-thin 2D nanomaterials, as a representative of graphene, exhibit unique physical and chemical properties because the electrons are confined in 2D space. These excellent properties make ultra-thin 2D nanomaterials enter the field of vision of researchers, ushering in the golden age of rapid development [5–11]. Since the discovery of graphene, more and more ultra-thin 2D materials have been found and synthesized, including hexagonal boron nitride (h-BN), carbon nitride (g-C_3_N_4_), transition metal chalcogenides (TMDs), transition metal oxides (TMOs), transition metal carbides (TMCs), layered double hydroxides (LDHs), metal − organic frameworks (MOFs), phosphorene and other elemental 2D materials [12–15]. These materials not only enrich the types of ultra-thin 2D nanomaterials, but also show a variety of properties due to the differences in composition and structure, which provide sufficient impetus for the follow-up research of ultra-thin 2D nanomaterials.

**Fig. 1:**
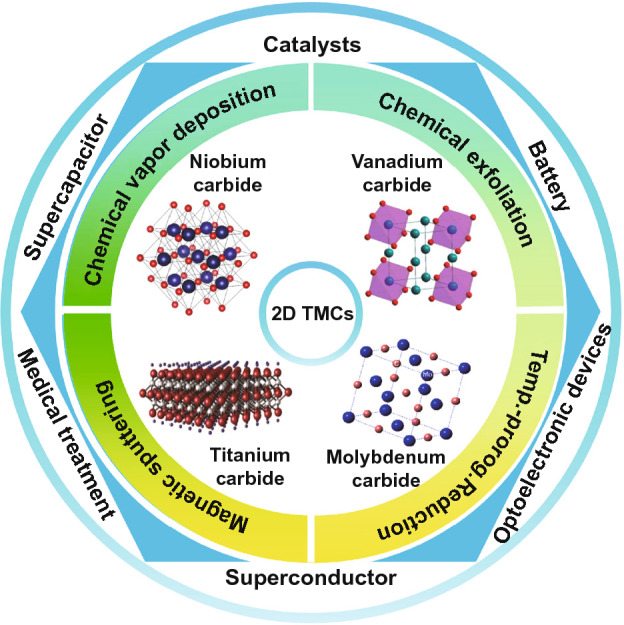
The scope of this review

In 2011, Gogotsi and Barsoum reported the synthesis of MXene, as a new member of 2D transition metal carbides (TMCs) [16]. The general chemical formula of MXene is M_n+1_X_n_T_z_ (*n* = 1, 2, 3), where M is transition metal element, such as Ti, Sr, V and Ta, X is C or N, and T stands for F-, OH- and other functional groups. So far, there are more than 70 members of the MXenes family reported. The MXene materials are typically prepared by selective etching the A layer (also named Al layer) with a high concentration of hydrofluoric acid using the MAX with layered hexagonal structure of ternary ceramic phase as the precursor [16]. Due to their 2D layered structure, good conductivity, stability, hydrophilicity and unique in-plane anisotropic structure, MXene materials have attracted many attentions in recent years. However, the fundamental properties, potential applications and even the controllably synthesis of TMCs are still in their early stage. Previous review progresses are mainly focused on the energy storage, especially on Ti-based TMCs (also called MXenes). The phase diagram, property and synthesis strategy of TMCs including Ti-based and other transition metal-based TMCs have rarely been overviewed. The scope of this review is shown in Fig. 1. We will introduce the structure, physical and chemical properties, the potential applications and finally, the preparation methods of typical TMCs including niobium carbide, vanadium carbide, molybdenum carbide and titanium carbide.

## Structure and Property of Transition Metal Carbides

In 1973, Levy and Boudart found that the carbon atoms in tungsten carbide would change the electron distribution of tungsten atoms resulting in the catalytic property similar with that of platinum and other precious metals [17]. This discovery led to extensive research on other early transition metal carbides, nitrides and carbonitrides. With the development of layered materials since 2004, the transition metal carbides (TMCs) recall its hot spot due to their many excellent dimensionality and structure-dependent properties. TMCs are mostly interstitial alloys formed by transition metal atoms and carbon atoms. Taking Mo-based TMCs as the example, the β-Mo_2_C and η-Mo_3_C_2_ of molybdenum carbide are orthogonal and the arrangement of Mo atoms in units is slightly different from that of hexagonal close-packed (hcp). The crystal structure can be described as that Mo atoms occupy the lattice site with the formation of hcp, while carbon atoms occupy half of the octahedral interstitial positions. However, the α-MoC_1-x_ has a face-centered cubic close-packed (*fcc*) crystal structure. In addition, Ti, Nb, V and Ta atoms in their TMCs all form *fcc* crystal structure [18]. These carbides are composed of two nested *fcc* lattices, one contains metal atoms and the other contains carbon atoms, which is similar to the NaCl crystal structure. The bonding configuration is usually formed through the hybridization between the 2 s and 2p orbitals of carbon atoms with the d orbitals of transition metals. With the increase in *sp* electrons, the parent metal structure gradually transfers from *bcc* crystal structure to hcp crystal structure, and then to *fcc* crystal structure. The lattice constant and bulk modulus of carbides have been calculated theoretically by Murnaghan equation, showing that the theoretical calculated values are in good agreement with the experimental values [19]. Recently, Frey et al. adopted a new model based on density functional theory-PU learning model, and studied 66 kinds 2D single transition metal atoms-based TMCs and there are more than 800 kinds of MAX with different phases through high-throughput calculation. The results predicted that about 111 kinds of MAX and 18 kinds of TMCs could be synthesized with the high possibility (Table 1) [20]. Specially, 14 of the 18 TMCs have the formation energies lower than 200 meV atom^−1^, which is below the threshold value, and the stability of 4 unstable TMCs (W_4_C_3_, Ta_2_C, W_3_C_2_ and Mo_4_C_3_) can also be improved by surface functionalization [21]. However, due to the low chemical activity and complex synthesis condition of these TMCs, until now, only the niobium carbide, vanadium carbide, molybdenum carbide and titanium carbide have get a reasonable investigated. Besides the existence of the TMCs, Table 2 exhibits the property of the typical layered materials, including the TMCs. One can see that the new layered TMCs have many excellent property, which would facilitate the development of the materials science. In the following, we introduce mainly the structure, synthesis, properties and applications of these four TMCs, which would inspire future studies.

**Table 1:** 18 kinds of MXenes with high synthesis possibility [20]

MXene predicted to be stable		
Hf_4_C_3_	Ta_4_N_3_	Sc_3_C_2_
Nb_3_C_2_	Ta_2_C	Ti_2_N
Zr_2_C	Hf_4_N_3_	Sc_2_C
Ta_3_C_2_	Ti_4_C_3_	W_3_C_2_
W_4_C_3_	Hf_2_C	Nb_2_N
Zr_4_C_3_	Sc_4_C_3_	Mo_4_C_3_

**Table 2:** Basic parameters of the typical layered materials

Materials	TMCs	Graphene	TMDs	BP	h-BN
Band gap (eV)	0 (Metallic)	0 (Metallic)	1.2 ~ 1.8 (MoS_2_)	0.3 ~ 1.5	6.07
Conductance (S/m)	~ 10^6^ (MXene fiber)	~ 10^6^	/	300	/
Critical temperature of Superconductor (K)	2 ~ 10 (Nb or Mo-based TMCs)	1.7 (Twist bilayer graphene)	12 (MoS_2_@130 GPa)	7.5 (@5 GPa)	/
Thermal conductance (Wm^−1^ K^−1^)	48.4 (Mo_2_C)	3000	52	4.3–5.5	300
Young’s modules (GPa)	14.0 (MXene)	1000	230 (MoS_2_)	20 ~ 100	1160
Stability in ambient	No	Yes	Yes	No	Yes

### Niobium Carbide

**Fig. 2:**
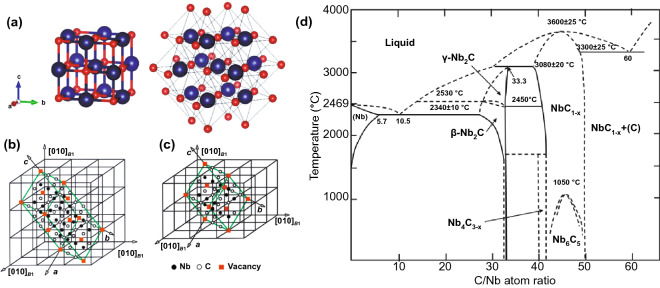
Structure of Nb-based TMCs. **a** Schematic diagram of the B1 type crystal structure of NbC. Reproduced with permission from Ref. [27]. Copyright 2016, MDPI. **b** NbC_0.38_ unit cell structure with C2 space group. **c** NbC_0.38_ unit cell structure with C2/m space group. Reproduced with permission from Ref. [25]. Copyright 2021, Springer Nature. **d** Phase diagram of C-Nb system. Reproduced with permission from Ref. [27]. Copyright 2016, MDPI

Among TMCs, niobium carbide has attracted much attention due to its excellent properties, such as high melting point (3610 °C), excellent chemical stability, high toughness, high Young’s modulus and higher hardness than other TMCs [22, 23]. Niobium carbide also exhibits better electrical properties, where its resistivity is as low as 4.6 μΩ cm at room temperature and will show superconductivity at 12 K [24]. Niobium carbide has a B1 type crystal structure (as shown in Fig. 2), and the vacancies only appear in the carbon sublattice. The composition of the ordered atom-vacancy crystal structure is close to NbC_0.38_ [25]. There are different solid single-phase zones in the NbC system: solid solution of carbon in niobium(bcc), γ-Nb_2_C, β-Nb_2_C, NbC_1-x_, Nb_6_C_5_, NbC and Nb_4_C_3-x_ [26, 27]. γ-Nb_2_C has a hexagonal structure and can be transformed into ordered hexagonal β-Nb_2_C at a lower temperature. NbC_1-x_ has a NaCl-type crystal structure and can be transformed into Nb_6_C_5_ with an ordered crystal structure at 1050 °C. NbC also has a NaCl-type crystal structure, which can be regarded as two *fcc* lattice structures interspersed with each other. The atoms in NbC have octahedral coordination, and carbon atoms occupy half of the octahedral gap. Nb_4_C_3-x_ is very similar to the ordered V_6_C_5_ crystal structure, but whether there is a stable Nb_4_C_3-x_ is still controversial. This phase may be produced by the peritectic reaction between NbC and Nb_2_C [27].

### Vanadium Carbide

**Fig. 3:**
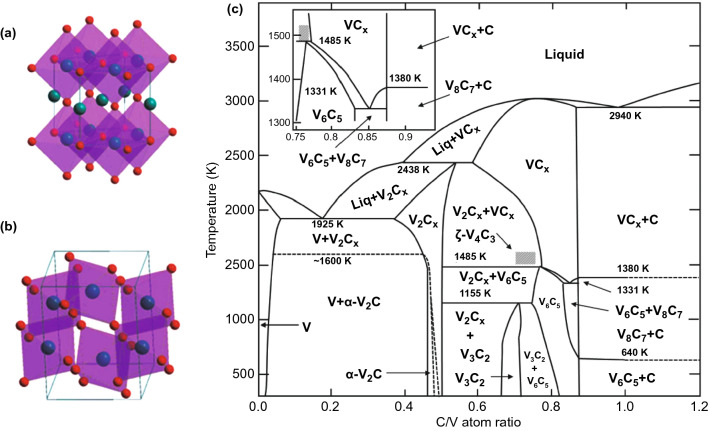
Crystal structure of V-based TMCs. **a** VC, **b** α-V_2_C. Reproduced with permission from Ref. [30]. Copyright 2011, The Royal Society of Chemistry. **c** Phase diagram of C-V system. Reproduced with permission from Ref. [31]. Copyright 1989, IOP Publishing

The vanadium carbide with stoichiometric composition (VC) cannot be obtained under equilibrium conditions. It usually has extensive homology disordered δ-VC_1-x_ (VC_0.65_-VC_0.90_) crystallizes with a NaCl cubic structure. Carbon atoms in NaCl-type vanadium carbides can only fill the octahedral vacancies of the metal *fcc* sublattice partially, that is, there are structural defects. Under certain conditions, their presence may lead to atomic ordering, which is caused by the redistribution of nonmetallic atoms and structural vacancies at interstitial lattice positions. Due to its high concentration of structural vacancies, this non-stoichiometric interstitial compound can be used in the field of electronic materials. It is found that the ordering of carbon atoms and the formation of structural vacancies in vanadium carbide are accompanied by the increase in micro-hardness and electrical conductivity [28]. Shacklete et al. studied the effect of ordered-disordered phase transition on the resistivity of vanadium carbide single crystal. The results show that the resistivity of vanadium carbide in disordered phase is significantly higher than that in ordered phase. There are 6 solid single-phase zones in the VC system: VC, α-V_2_C, β-V_2_C, V_4_C_3_, V_6_C_5_ and V_8_C_7,_ as shown in Fig. 3 [29]. Chong et al. systematically studied the stability, electronic structure and anisotropic mechanical properties of VC binary compounds by first-principles calculation, demonstrating their potential applications [30].

In the crystal structure of VC, each unit cell contains 8 atoms (4 V atoms and 4 C atoms). V_4_C_3_ is similar to VC, but each cell has one C atom vacancy and 7 atoms, including 4 V atoms and 3 C atoms. The appearance of natural carbon vacancy in V_8_C_7_ makes the space group become P43_3_2, and the maximum number of atoms in a unit cell is 60 (32 V atoms and 28 C atoms). But the structure of V_8_C_7_ is still cubic. The lattice parameters of V_4_C_3_, V_8_C_7_ and VC are 8.219, 8.315 and 8.305 Å, respectively. The lattice constant of V_4_C_3_ is less than VC, which should be due to the doping of carbon vacancies. On the other hand, the formation of natural carbon vacancies will change the space group in the actual V_8_C_7_ lattice, resulting in a slightly larger lattice constant [30]. The cohesive energy of VC binary phase increases in the following order: V_6_C_5_ < V_8_C_7_ < VC < α-V_2_C < V_4_C_3_ < β-V_2_C [30]. All vanadium carbides exhibit metallic property because of their narrow band gap at Fermi level. Near the Fermi level, the shape of the energy density curve of all VC compounds is similar to that of the V-d state, indicating that the d band of V atom dominates the Fermi level. The chemical bond of VC binary compound is mainly VC covalent bond, but it also has ionic and metallic properties, which makes vanadium carbide have a high melting point, high mechanical modulus, high hardness and good electrical conductivity [30]. Due to its high hardness, high melting point, excellent wear resistance, low friction coefficient and good corrosion resistance, vanadium carbide is often used to improve the life of mechanical components in tribological applications [32].

### Molybdenum Carbide

**Fig. 4:**
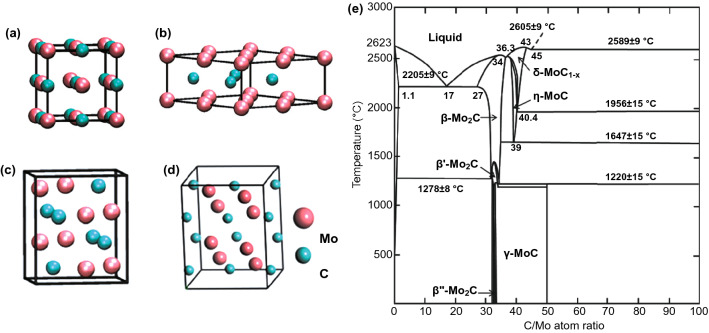
Bulk crystallographic structures of Mo-based TMCs. **a** fcc α-MoC_1−x_, **b** hexagonal γ-MoC and η-MoC, **c** orthorhombic β-Mo_2_C, **d** orthorhombic α-Mo_2_C. Reproduced with permission from Ref. [36]. Copyright 2018, Elsevier. **e** Phase diagram of C-Mo system. Reproduced with permission from Ref. [39]. Copyright 2001, American Physical Society

Molybdenum carbide has five different crystal structures: α-MoC_1−x_, α-Mo_2_C, β-Mo_2_C, γ-MoC and η-MoC [33]. For α-Mo_2_C, as shown in Fig. 4, two layers of Mo atoms are arranged in an AB structure, and a layer of carbon atoms is sandwiched in the middle, occupying octahedral center [34]. It is equivalent to that the Mo atoms are closely arranged in the hexagonal form and the carbon atoms are distributed in the octahedral gap with a Z-shaped structure. The lattice of Mo atoms is deformed because the carbon atoms deviate from the center of the gap, thus forming an orthogonal crystal structure. For β-Mo_2_C, the Mo atoms are arranged in a strict close-packed hexagonal form, and the carbon atoms still occupy 50% of the octahedral gap. Thus, the distribution of carbon atoms has a certain randomness [35]. The α phase is stable at room temperature, while the β phase is stable at high temperature and metastable at room temperature and can only exist stably above 1960 °C. Recently, Liu et al. reported that under the irradiation of electron beams, the carbon atoms in Mo_2_C would migrate resulting in that Mo_2_C change from α phase to β phase [35]. As regarding to the density of states of β-Mo_2_C and α-Mo_2_C [36], the total density of states is mainly composed of s, p orbitals of C and d orbital of Mo. Furthermore, there is no band gap near Fermi level indicating the metallicity of molybdenum carbide [36]. Molybdenum carbide has strong absorption for a large range of light, where the molybdenum carbide thin films have a uniform absorption in the range of 500–2000 nm [37]. Molybdenum carbide is a kind of saturated absorber. The absorptivity of molybdenum carbide to a certain wavelength decreases with the increase in light intensity. When the light intensity increases to a certain value, it is transparent to that wavelength. In this case, when molybdenum carbide is made into a grid with various periodic widths, and the grid is made into a transistor with MoS_2_ channels and electrodes, the device has a good response rate and a high light–dark current ratio in the range of 400–1400 nm [38].

### Titanium Carbide

**Fig. 5:**
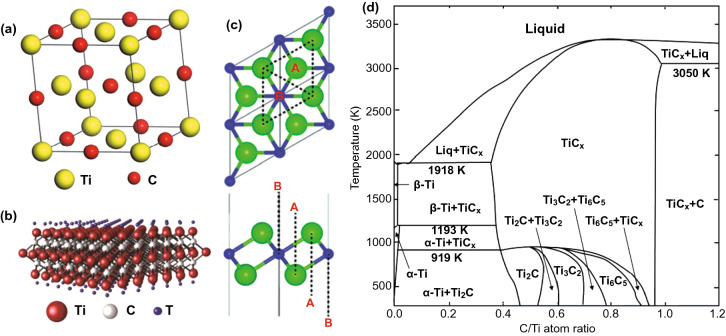
Structure of titanium carbide. **a** Ordered structures of cubic TiC. **b** Molecular structure model of single layer Ti_3_C_2_T_x_. Reproduced with permission from Ref. [46]. Copyright 2016, WILEY–VCH. **c** Molecular structure model of single layer Ti_2_CT_x_. Reproduced with permission from Ref. [47]. Copyright 2013, The Royal Society of Chemistry. **d** Phase diagram of C-Ti system. Reproduced with permission from Ref. [40]. Copyright 1997, Elsevier

Titanium carbide is a carbide with a wide homogeneity (from TiC_0.48_ to TiC_1.00_). The synthesis conditions will affect the ordered arrangement of vacancies in the carbon sublattice, leading to the appearance of non-stoichiometric TiC_x_, thereby resulting in the redistribution of carbon atoms and structural vacancies, and forming various ordered structures [40]. When the carbon vacancies are randomly distributed, the disordered TiC compound forms a cubic NaCl crystal structure. When the carbon vacancies are distributed in an orderly manner, there are two stable ordered titanium carbide phases, one is the cubic phase and the other is the triangular phase [41]. TiC with NaCl cubic crystal structure (as shown in Fig. 5) is the most common phase of titanium carbide and has been widely studied. The results show that the lattice spacing of ordered cubic phase Ti_2_C (space group Fd3m) is twice as large as that of disordered titanium carbide [42, 43]. The Ti_6_C_5_ phase is a stable ordered phase, and also a non-stoichiometric ordered phase of all IV and V group transition metal carbides [40]. Khaenko et al. demonstrated the existence of rhombohedral Ti_8_C_5_ [44]. Through grinding and polishing of titanium carbide, Dzhalabadze et al. found that 6H-type ordered titanium carbide with *fcc* lattice was formed on the surface [45]. In the process of the deposition of diamond films on titanium alloy substrate, Li et al. also found 6H-type titanium carbide at the interface [41].

In 2011, MXene, represented by Ti_3_C_2_T_x_, entered the field of vision of researchers, and the research on titanium carbide rose again and expanded to various fields. The structural study of titanium carbide based on Ti_3_C_2_T_x_ and Ti_2_CT_x_ MXene is also carried out gradually (as shown in Fig. 5). Through the heating treatment of Ti_3_C_2_T_x_ MXene, it was found that Ti_3_C_2_T_x_ has been significantly transformed into cubic TiC at 1100 °C. As the temperature increases, free C is lost due to the conversion of CO_2_/CO, and holes appear in the accordion layered structure. When the heat treatment temperature reaches 1250 °C, Ti_3_C_2_T_x_ MXene completely transforms into cubic TiC [44].

## Synthesis of Transition Metal Carbides

Although TMCs exhibit many excellent properties, the controlling synthesis is still in its infant. There are a few issues that need further investigation. During the synthesis of TMCs, the production of surface pollutants will block the active sites and cavities, resulting in a suppressed electrocatalytic activity. Normally, the traditional preparation methods of TMCs are usually based on solid–solid reaction or gas–solid reaction, that is, the directly pyrolysis of metal carbonyl compounds or the reaction of metal/metal oxides with C source. However, at relatively high temperature, the aggregation or overgrowth of TMCs during pyrolysis leads to the decrease in electrochemical reaction active sites and electrocatalytic activity. So far, researchers have been committed to enhance the electrocatalytic activity through the engineering of structures and interfaces, including nanostructures, doping, morphology controlling and the introduction of various carbon-based materials. To sum up, the reasonable design of the preparation process is essential to maximize the exposure of the active sites of TMCs in the process of efficient electrochemical reaction [48].

Besides the particle-like TMCs utilizing in catalytic and energy storage fields, film is another fashion of TMCs, where the solid materials have tiny dimensions in one dimension. Because the thickness is small, the proportion of surface particles is large, and the continuity of the structure is restricted by the surface interface, the properties of the thin films are quite different from those of the bulk materials including [49]: the decreases of melting point [32]; the selective projection and reflection of light [50]; the generation of surface energy level and surface magnetic anisotropy [51]; the varied critical temperature of superconductivity [52]; and the generated tunnel current in the direction of the thickness [53]. With the dimensionality decrease from three-dimensional to two-dimensional, the few-layered graphene and MoS_2_ display many unique properties, which are quite different with their bulk states, such as higher carrier mobility and field modulated effect [53, 54]. Due to the high melting point of most carbides, the TMCs materials can be hardly prepared by directly thermal evaporation, while electron beam evaporation has been used in the preparation of TiC/TiB_2_ films [49]. Up to now, some typical methods have been used to prepare the TMCs.

### Chemical Exfoliation

**Fig. 6:**
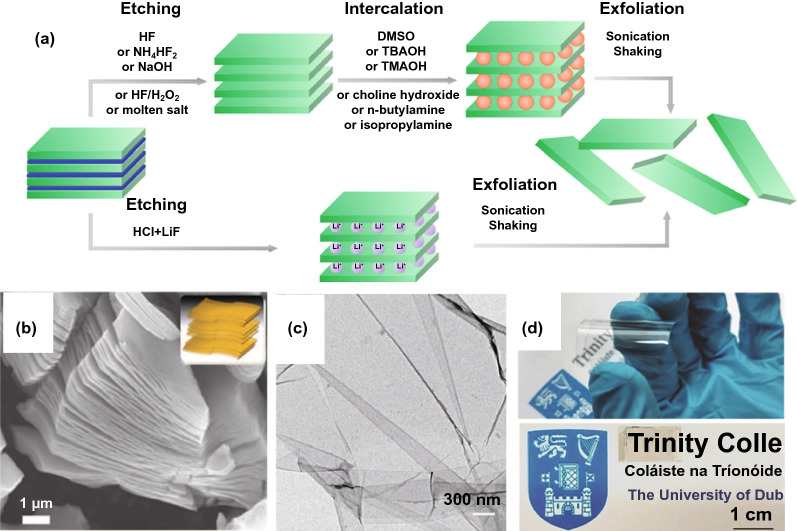
MXene preparation by chemical exfoliation. **a** Mechanism of chemical exfoliation. Reproduced with permission from Ref. [56]. Copyright 2019, Elsevier. **b** SEM image of intercalated procure. Reproduced with permission from Ref. [57]. Copyright 2013, The American Association for the Advancement of Science. **c** TEM image of few-layer MXene. Reproduced with permission from Ref. [58]. Copyright 2014, Royal Society of Chemistry. **d** Digital image of MXene-based transparent electrode. Reproduced with permission from Ref. [59]. Copyright 2017, WILEY–VCH

**Fig. 7:**
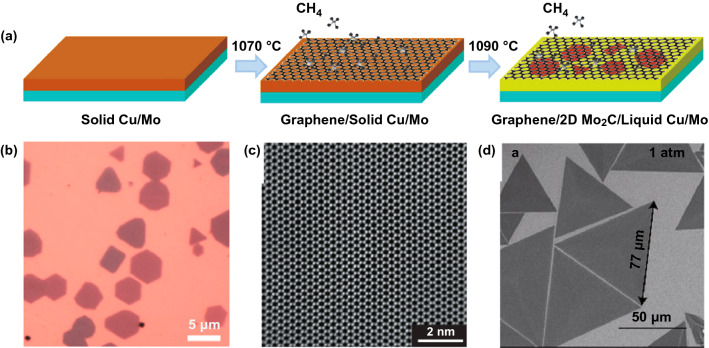
Preparation of TMCs by chemical vapor deposition. **a** Mechanism of Mo_2_C growth, where the Cu foil is located on the surface of Mo foil. Reproduced with permission from Ref. [95]. Copyright 2017, American Chemical Society. **b** Formation of Mo_2_C flakes under graphene under high temperature. Reproduced with permission from Ref. [95]. Copyright 2017, American Chemical Society. **c** Atomic pattern of Mo_2_C by high resolution TEM. Reproduced with permission from Ref. [90]. Copyright 2015, Springer Nature. **d** SEM image of VC flakes. Reproduced with permission from Ref. [96]. Copyright 2020, Elsevier

Chemical exfoliation is that it uses HF and LiF to selectively etch the A layer of the parent phase MAX with three-dimensional layered structure to realize the preparation of carbides, nitrides and carbonitrides, where M is transition metal (Cr, Ti, V, Cr, Zr, Nb, Mo, Hf or Ta), A is mostly IIIA or IVA group elements (Al, Si, etc.), and X is C or N [55]. In order to highlight the similarity between the product and graphene, Naguib et al. named it with MXene. At present, the general process of synthesizing MXene by chemical exfoliation mainly includes: Max precursor synthesis, etching and exfoliation. The schematic diagram of the process of preparing MXene by chemical exfoliation is shown in Fig. 6 [16]. Taking Ti_3_AlC_2_ as an example, Naguib et al. reported a method to remove Al layer from Ti_3_AlC_2_ without destroying the layered morphology. It was found that the exfoliated Ti_3_C_2_ with large interlayer distance can be obtained by treating Ti_3_AlC_2_ powder with 50% HF aqueous, and then, few-layered Ti_3_C_2_ flakes can be prepared through ultrasonic treatment in methanol [16] (Fig. 7).

Many aluminum-based MAX phases are synthesized at a temperature above 1300 °C [60]. Most of the M-A bonds in the layered MAX precursor phase are metal bonds or covalent bonds, which rules out the possibility of producing MXenes by mechanically shearing their parent phase MAX. Element A can be selectively etched using electrochemical reactions which take place in an acidic solution or an alkaline solution [61]. Recently, another type of layered solids has also been used as precursors, which is (MC)_n_Al_3_C_2_ and (MC)_n_(Al, Si)_4_C_3_; for example, Al_3_C_3_ and (Al, Si)_4_C_4_ were etched to obtain Zr_3_C_2_T_x_ and Hf_3_C_2_T_x_ [62]. However, except for Ti_3_SiC_2_, only the Al-containing MAX phase was successfully etched to synthesize MXene. The experiments also show that the MAX phase with larger n atom and larger M atom mass often requires longer etching time and more corrosive solution, which may be due to the large number of M valence electrons [63]. Etching conditions usually depend on the chemical structure of the parent phase. For example, if 50 wt% HF is used for etching Ti_2_AlC and Cr_2_AlC, the sample will complete dissolute. Although Ti_2_CT_x_ can be obtained by reducing the concentration of HF to 10 wt%, it still not works on Cr_2_AlC [64].

In order to avoid or minimize the use of concentrated HF due to its very strong corrosive, a few other synthetic pathways have been proposed. One of the most widely methods uses a mixture of hydrochloric acid (HCl) and fluoride salts. Using fluorides (LiF, NaF, KF and NH_4_F) and HCl solution, it was found that the Ti_3_AlC_2_ can be effectively etched and exfoliated to produce layered Ti_3_C_2_ MXene [64–67]. Alhabeb studied the etching effect under different molar ratio of HCl to LiF/HCl, and found that the MXene obtained by chemical exfoliation includes Ti_2_CT_x_, Ti_3_C_2_T_x_, V_2_CT_x_, etc., where T_x_ is a surface atom or atomic group, such as O, OH and F [68]. The surface hydrophilicity, conductivity and other physical and chemical properties of MXene prepared by etching method have a great relationship with the choice of etchant and the process. For example, etching with HF will make the surface of MXene mainly contain fluoride functional groups, but LiF-HCl etching will make MXene surface with oxygen-containing functional groups [69].

Similar to titanium-based TMCs, vanadium-based TMCs can also been prepared through this chemical exfoliation [70, 71]. He et al. used the mixture of NaF and HCl as an etchant to chemically strip V_2_AlC. The obtained layered V_2_C MXene has a high specific surface area of 19.3 m^2^ g^−1^ [72]. Zada et al. proposed a new chemical stripping method for large-scale preparation of MXene, which proves that the algae extract can effectively intercalate and strip V_2_AlC, avoiding the use of traditional HF and other dangerous etchants, and has the advantages of environment friendly and low cost [50]. Up to now, the main method to prepare V_2_C MXene is chemically etching. However, the formation energy of V_2_C from V_2_AlC is relatively high, the complete removal of Al layer in V_2_AlC is difficult, and thus, the final V_2_C MXene usually contains a certain amount of unreacted V_2_AlC. Therefore, the conversion efficiency of V_2_AlC to V_2_C needs to be improved, which is of great significance for the further application of V_2_C MXene [73]. Guan reported that the purity of V_2_C MXene can be up to 90% when using the mixed solution of LiF and HCl to treat V_2_AlC [74]. The Zr- and Nb-based TMCs have also been synthesized. Zhou synthesized two-dimensional Zr_3_C_2_ MXene by the similar chemical exfoliation using layered Zr_3_Al_3_C_5_ as parent phase MAX. It was found that Zr_3_C_2_ MXene has better structural stability at high temperature, compared with that of Ti_3_C_2_ MXene, suggesting its potential advanced application [62]. Xin studied the effect of surface functional groups on the work function of Nb_n+1_C_n_ MXene through density functional theory. The results show that the terminated F and O atoms will increase the work function of Nb_n+1_C_n_ MXene, while the OH and OCH_3_ groups will decrease its work function, indicating its widely potential application in electronics [75, 76]. Pang used a new, fluorine-free, concise and rapid synthesis method to prepare one-dimensional Nb_2_CT_x_ nanowires. The synthesis process includes a two-step etching process: the first is hydrolysis, and the second is 3D electrode thermally assisted electrical etching. With strong stirring, the parent phase MAX creates gaps on the TiC surface and splits into small pieces, the lateral size of which is reduced from 10 ~ 30 to 1 ~ 5 μm. Under ultrasonic treatment, a shorter etching time can make the MAX-MXene composite produce nanowire “shred effect” [77].

The chemically exfoliation has also been used to synthesize the multi transition metal-based TMCs. Pinto et al. prepared the two-dimensional bimetallic TMCs (Mo_x_V_4-x_C_3_ MXene) by selectively etching Al from the Mo_x_V_4-x_AlC_3_ precursor. Unlike the reported ordered bimetallic carbides Mo_2_TiC_2_ MXene and Mo_2_Ti_2_C_3_ MXene, the Mo and V layers in this Mo_x_V_4-x_C_3_ MXene exist in the form of solid solution. By changing the precursor composition, four different types of Mo_x_V_4-x_C_3_ MXene with x = 1, 1.5, 2 and 2.7 have been obtained [78]. However, due to the difficult synthesis of stable MAX precursors, many predicted TMCs MXenes have not been successfully synthesized. For example, MAX precursors for Cr_3_N_2_, Mo_3_N_2_, Hf_3_N_2_ and Cr_3_C_2_have not been reported. On the other hand, even some MAX phase can be synthesized; the chemically exfoliation also faces challenge because the as-prepared TMCs MXenes also can be destroyed and solved in the hydrofluoric acid aqueous. For example, although the MAX phase of Cr_2_AlC was synthesized long time before, the Cr_2_C MXenes was yet well prepared [79]. It was found that the samples can be dissolved after a few hours even the etchant concentration has been greatly diluted [80].

Except the etchant, the used dispersion solution is also important, which can affect the size and the stability of the exfoliation flakes. It was found that the yield is quite low when directly stripped by ultrasound in etchant [81, 82]. Recently, it was reported that Ti_3_C_2_ MXene and (Mo_2/3_Ti_1/3_)_3_C_2_ MXenes can be stripped by polar organic molecule dimethyl sulfoxide (DMSO) [83, 84], but it has no obvious effect on other TMCs-based MXene. Tetrabutylammonium hydroxide (TBAOH), which is commonly used for stripping other two-dimensional materials [85], has also been successful in stripping V_2_C MXene and Ti_3_CN MXene with good exfoliation and stability [86]. Nb_2_C MXene can also be stripped in isopropylamine [87].

In summary, the etchant solution and the dispersion solution play the key role in the chemical exfoliation. Although using the fluoride salts can somehow decrease the dangerous of the protocol, the yield and the size of the MXene flakes are still need further improve. The dispersion solution is another important factor, which not only affects the exfoliation rate, but also affects the stability of the as-prepared MXene, indicating that more efforts are still needed.

### Chemical Vapor Deposition

Chemical vapor deposition (CVD) is another method which can grow film with large scale and high quality, thus has been widely used in industry. To grow film by CVD, the sources are usually supplied with the formation of gas, which can be easily controlled. However, recently, considering the rare and expensive gas source of transition metal, the traditional CVD has been modified; for example, the gas source of transitional metal was supplied by pre-heating its corresponding transition metal oxide. By this modified CVD method, the high crystallinity MoS_2_ films have been grown [88, 89]. The products obtained by this method are very different from those obtained by chemical exfoliation, where the source molecular would react and deposited on the growth substrate. Under high temperature, the molecular or cluster of the samples would migrate and re-organized to form film with high quality, such as singe-crystal-like film or flakes. Therefore, its fashion is quite different from the sample prepared by chemical exfoliation and has yet named with MXene.

In 2015, Xu et al. reported for the first time the growth of high-quality ultra-thin TMCs crystals (Mo_2_C superconducting crystals) by CVD method using double-layer metal foils (copper, copper/transition metal) as substrates; By these methods, Xu et al. also grown TaC and WC thin films [90]. Firstly, the Cu/Mo foil laminate was heated to above 1085 °C (Cu melting point) in hydrogen, and then, the Cu metal would melt and form a uniform liquid Cu film on the Mo substrate. Methane was introduced at a low flow rate to form Mo_2_C crystals on the surface of the liquid Cu. The top liquid copper layer plays an important role in the growth process. On the one hand, it acts as a catalyst to decompose methane into carbon atoms. On the other hand, it acts as a channel to control the diffusion of Mo atoms from the Mo foil to the surface of the liquid Cu. The results show that ultra-thin Mo_2_C crystals are formed on the surface of Cu by the reaction of C atoms and Mo atoms. Once the growth is finished, Mo_2_C can be further transferred to any target substrate by etching Cu, which is similar with the transferring of graphene [91]. However, it should be noted that the thinnest Mo_2_C film is composed of at least six layers of Mo_2_C rather than a monolayer film, suggesting that the growth of monolayer film needs further optimization [90].

Geng et al. reported an one-step directly growth of Mo_2_C where its size can be grown as large as centimeter [92]. Through controlling investigation, it was found that there is no graphene layer formed with low methane flax. However, at higher methane flax, the graphene would firstly form on the surface of liquid Cu, and the migrated Mo atoms would go through the graphene layer and form the Mo_2_C cluster on the surface of graphene. Thus, the underlayer graphene would work as a buffer layer during the growth and guild the further growth of Mo_2_C crystal with its preferable morphology. Furthermore, the graphene layer would also block the migration of Mo atoms, resulting in the thin of Mo_2_C crystal (about 8.32 nm). If there is no graphene layer, the as-grown Mo_2_C crystal can be as thick as 237 nm. In addition, the thickness of Mo_2_C crystal can be tuned by varying the thickness of cooper layer, where the kinetics of Mo diffusion across the Cu layer can be modulated. The thinnest Mo_2_C crystals with thickness of 9.5 nm corresponding to 20 layers were obtained [93]. With the similar method, Zhang et al. placed the V foil, Cu foil and W foil in order. At higher temperature, the Cu foil would be melted on the surface of W foil; however, the V foil is still solid state. By controlling the temperature and methane flax, the VC flakes can be obtained on the surface of Cu foil and its thickness is about 12 nm [94]. By increasing the flux of hydrogen flax, it was found that the morphology of VC crystal would evaluate from continue film to branch shape, suggesting the etching role of hydrogen. Interestingly, although the W foil was employed as the substrate, there was no WC crystal formed. As a comparison, the researchers also found that there are no crystal-like VC flaks but only dense VC polycrystalline film formed when using Cu foil/V foil as the source, due to the large amount of migrated V ions on the Cu surface.

Ikenoue et al. prepared the uniform WC_1-x_ film on the substrate surface by mist CVD method, where the WCl_6_ acetonitrile solution was carried into the furnace by Ar/H_2_ mixture gas. When the temperature is higher than 650 °C, WC_1-x_ begins to form, and with the increase in preparation temperature, the element ratio of C/W is gradually close to 1. Mechanical characterization shows that the hardness and Young’s modulus of WC_1-x_ films grown at 750 °C are 25 and 409 GPa, respectively [97]. Atomic layer deposition has also been used to prepare NbC thin films by employing NbF_5_ and NbCl_5_ as the raw materials, TMA as a carbon source and reducing agent. The NbC film is amorphous with a thickness of about 60 ~ 70 nm. If the film is thicker, NbC nanocrystals with a diameter of 15 nm will be formed. SQUID magnetometer measurements show that the 75-nm-thickness NbC film displays superconducting behavior where its transition temperature is about 1.8 K [52].

Carbide films with different compositions which synthesized by CVD, such as Ti (C_x_N_y_), TiC/TiN, TiC/Al_2_O_3_, TiC/TiB_2_ and TiC/Al_2_O_3_/TiN multilayer films, have been developed and applied [98]. However, some problems have been found in the process of gas-phase synthesis: the synthesized carbides are usually polluted by the pollutant produced by the pyrolysis of carbonaceous gases. The pollutants block the pores, wrap the active sites on the surface of the carbides, which are difficult to be eliminated. In addition, most gas-phase synthesis processes are not only tedious and complex, but also involve the use of expensive and toxic reagents, such as gaseous molybdenum precursors, which are harmful to organisms and the environment. Thirdly, the current reaction toward single-crystal TMCs film is usually carried out under the assistant of Cu and high temperature. Considering the plasma or laser treatments may help to improve the activity of transition metal or the carbon source, the growth temperature may decrease such as growing by plasma-assisted chemical deposition or laser-assisted chemical deposition. In addition, the development of the transition metal-based organic gaseous precursor may help to grow the TMCs film by metal–organic chemical vapor deposition, which can further optimize the growth condition.

### Temperature-programmed Reduction

To improve the catalytic property of TMCs, one of the strategies is that synthesize TMCs with high surface area. With the developing, some methods have gradually developed, including gas-phase reactions that occur using gaseous precursors of metal compounds, reactions between gaseous reactants and solid metal compounds, and thermal decomposition of metal precursors. Among them, the temperature-programmed reduction (TPR) developed by Boudart et al. has a broad prospect [99]. So far, almost all the work has been focused on the synthesis of molybdenum and tungsten based carbides, and few other transition metal carbides have been studied. However, TPR has broad research space in the synthesis of binary and ternary early transition metal carbides used in the field of catalysis, due to its easily synthesis condition [100]. It was found that below 1500 K, the mixture of Mo and C has the four phases: Mo, β-Mo_2_C, α-MoC_1-X_ and C, which depends on the relative content of the two components [101]. Through the study of Teixeira, the synthesis temperatures are 1170 K for both NbC and vanadium carbide [102–104]. However, to synthesize the TaC, the temperature should increase to 1220 K [105]. Directly carbonizing metal has also been studied, where a W/C (10 nm/20 nm) planar heterostructure was pre-prepared by magnetron sputtering. Then, the original crystallization of W/C heterostructure was studied in the temperature range of 300 to 1200 °C. It is found that the nucleation process of reactive synthesis of metal carbides is realized by two-step mechanism. Firstly, the amorphous intermediate with spinodal structure is formed by an amorphous precursor, and then, nucleation of amorphous intermediate occurs [106].

Besides the transition metal was used as the source, John et al. demonstrated that the TMCs can also be synthesized by employing vanadium, niobium, tantalum, molybdenum, tungsten and other binary or ternary oxides as transition metal raw materials. During the TPR process, it seems that it is easier to synthesize carbides for the ternary oxides of V group and VI group, because either the reaction rate is faster or the synthesis temperature is lower [100]. Post-annealing treatment has also been carried out. After post-annealing at 1500, 1600, 1700 and 1800 °C for 2 h, it was found that the lattice constant and average grain size of ZrC increased. The crystal grain orientation changed and the crystallinity of ZrC increased with the annealing temperature; at the same time, the structural defects decreased and the hardness decreased slightly [107]. Sun et al. have synthesized high-quality and high-density TaC through the high-pressure high-temperature (HPHT) sintering method. Under a pressure of 5.5 GPa, the pre-compressed TaC powder is heated to 1400 °C with a temperature increase rate of 150 °C min^−1^. After holding for 20 min, the sample was quenched to room temperature at a cooling rate of 150 °C min ^−1^. The Vickers indentation test shows that the TaC sample has a mechanical strength of 20.9 ± 0.5 GPa, which is about 35% higher than the reported data [108].

**Fig. 8:**
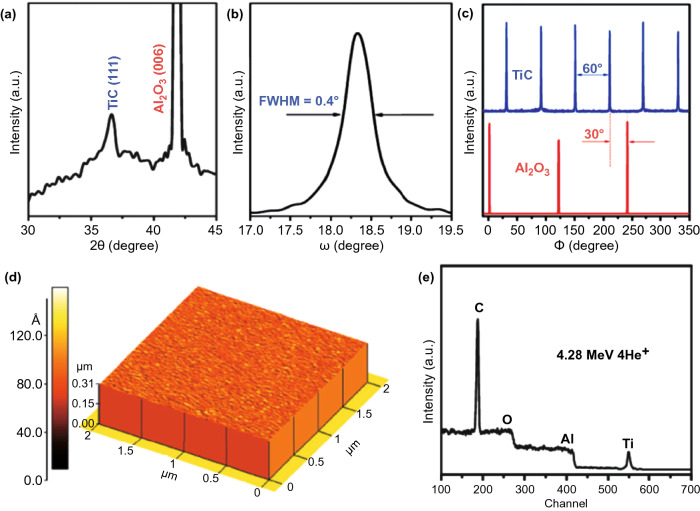
Preparation of TiC by temperature-programmed reduction. **a**-**c** XRD analysis of TiC film. **d** AFM morphology of TiC film. **e** Element analysis of TiC film. Reproduced with permission from Ref. [109]. Copyright 2010, American Chemical Society

To prepare the TMCs film by the TRP, polymer-assisted deposition (PAD) has been employed where the transition metal ion would first bind with polymer. Zou et al. prepared the Ti, V or Ta precursor by binding these ions with EDTA and PEI polymer, as shown in Fig. 8 [109]. Through annealing the spin-coated precursor, the TiC, VC and TaC films have been prepared. Especially, it was found that the as-prepared TMCs film has high quality and epitaxial on the sapphire substrate, where its grain size and roughness are 50 and 3.5 nm, respectively. Further studying shows that the hardness and Young’s modulus of TiC films are 21.27 and 413 GPa. The TiC film shows a semiconducting behavior, where its resistivity at room temperature is about 372 μΩ cm. By the similar method, the uranium dicarbide films have also been epitaxially grown on yttria-stabilized zirconia substrate [110]. The investigation exhibits that by controlling the precursor and the annealing progress, it was able to prepare TMCs film with high quality. Considering the low cost and high yield of spin-coating technology, this method can grow TMCs with large scale. However, controlling binding the transition metal ions with polymer is still a challenge, the binding rate needs further optimization.

### Magnetic Sputtering

Magnetic sputtering is another kind method to grow film with large scale, which has also been utilized to grow TMCs films. Due to the high wear resistance, conductivity, hardness and oxidation resistance, niobium carbide has been well studied than other TMC materials [23, 111–113]. By DC reactive magnetron sputtering using pure Nb target, the effects of deposition rate, chemical bonding, phase composition, microstructure and internal stress on the properties of the NbC_x_ films have been studied [23, 114]. The results show that the hexagonal Nb_2_C phase would form when the carbon content is 32.7 at%. However, cubic NbC phase with a mixed orientation of (111) and (200) would form when the carbon content is higher than 32.7 at%. Thus, by tuning the carbon content, the phase can be varied between hexagonal Nb_2_C and cubic NbC. Considering the hexagonal Nb_2_C phase has higher hardness than that of cubic NbC phase [115, 116], the Nb-based TMCs film with tunable hardness can be prepared by tuning the carbon content. In addition, when the carbon content is varied from 41.8% to 68.7%, the grain size would decrease monotonically from 40.6 to 3.9 nm [114]. Molybdenum carbide film has also been grown by radio frequency magnetron sputtering by using Mo_2_C target, and its application in the generation of solid-state passive Q-switched pulsed lasers has been studied [117]. At 1064 and 1342 nm, the Mo_2_C films show a large nonlinear saturated absorption, and the modulation depth is 10.39% and 8.89%, respectively, suggesting a well broadband nonlinear optical application.

By magnetic sputtering, the TMC film with large scale can be grown facilitating its potential application; however, the technology has not well investigated. More efforts may input to study its crystallization and texture; the additional carbon sources also need to be considered to improve the carbon vacancy.

## Application of Transition Metal Carbides

### Electrocatalysis and Photocatalysis

**Fig. 9:**
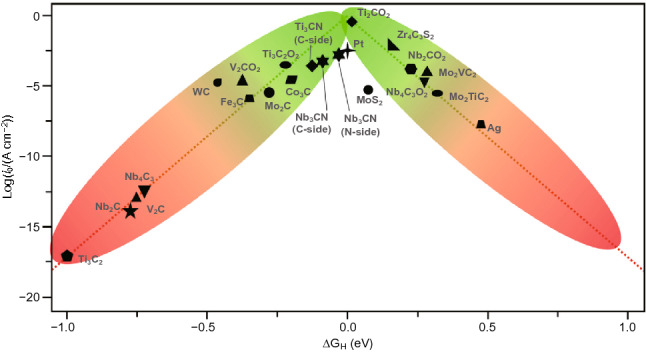
Exchange current as the function of Gibbs free energy of hydrogen adsorption of TMCs

At present, precious metals such as platinum (Pt), palladium (Pd) and rhodium (Ru) have shown favorable activity toward hydrogen evolution reaction (HER). However, the application of these precious metals is greatly hindered because of their low abundance and high cost. TMCs with high abundance in the earth, such as Ni_3_C, Mo_2_C and VC, have been proved to be excellent catalysts for HER both theoretically and experimentally. Most of the previous studies on TMCs were conducted on low surface area materials. However, the key to the preparation of high efficiency catalyst lies in the synthesis of high surface area materials [118]. Theoretical calculation shows that TMCs meet the basic requirements of hydrogen evolution reaction (HER). In fact, TMCs (such as Ti_2_C, V_2_C and Ti_3_C_2_) with -OH and -O on their surface are the basis of their metallicity, which causes charge transfer and transport. In addition, oxygen atoms which on the surface of TMCs provide active sites for HER, because the interaction between O atoms and H atoms on the surface of TMCs promotes the removal of hydrogen [119–121]. The volcano curve reflects the ability of various TMCs for HER in Fig. 9. TMCs at the top of the volcano have the highest catalytic activity, such as Ti_2_CO_2_, W_2_CO_2_, TiVCO_2_ and Nb_2_CO_2_. In addition, bimetallic TMCs (M_1_M_2_CO_2_) are also potential candidates because they have moderate H_2_ adsorption free energy catalysts for HER, thus showing higher activity. For these reasons, TMCs-based systems have become a hot spot in the design of electrocatalysts and solar-powered photocatalysts [122].

Using the adsorption of atomic hydrogen as a probe, the chemical properties of the surfaces of different carbide can be studied. Due to the tensile strain generated on the carbide surface when carbon is bonded to the crystal lattice, the adsorption of hydrogen to the carbides surface which at the end of metal is stronger than that to the tightly filled pure metal surface. John et al. found that the adsorption of hydrogen atoms on the Mo terminated surface of molybdenum carbide is much stronger than that on the surface of pure metal Mo (110) [19]. Compared to the ground-state pure metal surface, the metal terminated surface of the carbide has the lower hydrogen bonding energy (HBE) values. It can be seen that for other carbides except VC, the adsorption of H on the terminated surface of TMCs is stronger than that on the surface of pure metal. One of the reasons for the strong adsorption energy of the TMCs surface may be that the carbide surface is in a state of tensile strain compared with the pure metal surface. The distance between metal atoms in TMCs is farther than the distance between metal atoms in pure metal [19].

Wan et al. have systematically analyzed the crystal structure, electronic properties, free energy, surface energy and crystal formation energy of V_4_C_3_, V_8_C_7_ and VC_3_ during HER and OER processes by using the first-principles calculation method [123]. The results show that the vanadium carbide has excellent HER performance but poor OER activity. In particular, V_8_C_7_ has the best HER activity in these vanadium carbide phases. Compared with other phases, V_8_C_7_ has excellent catalytic activity, which can be attributed to the following factors: (i) larger surface energy is easier to capture ionized hydrogen/oxygen; (ii) more moderate hydrogen adsorption energy can accelerate HER rate; (iii) lower crystal formation energy and easier formation of C defects increase the specific surface area and active center of HER, and provide faster charge transport for HER; (iv) larger VC bond length and weaker bond strength contribute to the formation of suitable hydrogen absorption energy and smaller free energy ΔG (H*). In addition, there is a significant similarity in the density of d-band states between VC/V_8_C_7_ and Pt on the (110) and (111) crystal planes, indicating that the HER mechanism of VC/V_8_C_7_ is similar to Pt [123].

Experimentally, Tian et al. found that the combination of vanadium carbide and TiO_2_ can be used as an effective and stable co-catalyst for photocatalytic hydrogen evolution [124]. As a co-catalyst, VC can not only effectively capture the photogenerated electrons from TiO_2_, greatly improve the separation efficiency of photogenerated charges, but also significantly reduce its overpotential, thus enhancing the catalytic activity of TiO_2_/VC. Besides, the vanadium carbide has also be hybridized with active metal nanoparticle, where Pt nanoparticles with an average particle size of 3 nm are evenly distributed on the surface of carbon and cubic vanadium carbide (Pt/VC-C), which can be used as an electrocatalyst for oxygen reduction reaction (ORR) [125]. The combination of Pt nanoparticles and cubic vanadium carbide nanoparticles is beneficial to enhance the synergistic effect. Compared with the reversible hydrogen electrode (RHE), the mass activity of ORR on the surface of Pt/VC-C can reach 230 mA mg^−1^_Pt_ at 0.9 V, which is 2.4 times higher than that of Pt/C electrocatalyst (97 mA mg^−1^_Pt_). Furthermore, the vanadium carbide has also been demonstrated to effectively encapsulate on carbon-based skeleton, delivering a great HER activity such as a current density of 100 mA cm^−2^ and an overpotential of 238 mV [126]. Yoon et al. successfully doped V_2_CT_x_ with controllable concentration of phosphorus. The experimental results are in good agreement with the theoretical calculation that the P–C bond in P-V_2_CT_x_ works as active sites promoting the weakening of the hydrogen bond strength and leads to the desorption of H_ads_ during the HER process. V_2_CT_x_ with the highest P–C bond concentration exhibits a Tafel slope of 74 mV dec^−1^ and an overpotential of 163 mV at 10 mA cm^−2^ [127, 128].

Molybdenum carbide has been widely employed as electrocatalyst to split water, as shown in Table 3. Chen et al. studied the formation of molybdenum carbide from ammonium molybdate in inert environment. It was found that the coupling effect caused by the covalent bond between Mo_2_C and carbon carrier has a unique effect on the electrochemical performance. First of all, the conjugation with high bond strength can promote the close combination of Mo_2_C catalyst and carbon carrier, and provide a low resistance path suitable for rapid electron transfer. Secondly, this binding hinders the aggregation of Mo_2_C nanoparticles, thus promoting the production of highly active sites on the surface. Third, anchoring induces the transfer of charge from molybdenum to carbon, which further reduces the d-band center of molybdenum, thus reducing the hydrogen bonding energy of molybdenum. This, in turn, is beneficial to the electrochemical adsorption of H_ads_, resulting in a relatively moderate Mo-H bond binding strength, which enhances the HER performance. Molybdenum itself is considered to be a strong hydrogen-bonded metal due to its unique d-band position [129, 130]. To further improve the activity and the amount of active sites, doping heteroatoms has been considered. After boron doping [131], the HER activity of Mo_2_C catalyst is significantly improved, where the slope of Tafel downs to 78 mV dec^−1^, which is much smaller than that of the blank control (134 mV dec^−1^). In addition, the nitrogen-doped WC nano-arrays also show excellent HER activity, where overpotentials were 89 and 190 mV corresponding to the current density of 10 and 200 mA cm^−2^, respectively. Furthermore, the initial potential of the water splitting is 1.4 V when employing N-WC nano-array as both the cathode and anode, suggesting its high activity [132]. This is mainly due to the increase in the number of active sites, the turnover frequency increases, and the resistance to electron transfer decreases.

**Table 3:** Electrocatalytic performance of transition metal carbides

Sample	Prepare method	Morphology	HER or OER	Tafel slope (mV dec^−1^)	Overpotential at 10 mA cm^−2^ (mV)	Refs
Ti_3_C_2_	Chemical exfoliation	Nanofibers	HER	97 (H_2_SO_4_)	169	[7]
Ti_2_CT_x_	Chemical exfoliation	Nanosheets	HER	100 (H_2_SO_4_)	75	[138]
W_2_C	Microwave combustion	Nanodots	HER	45 (H_2_SO_4_)	71	[139]
Mo_2_C	Microwave combustion	Nanodots	HER	46 (H_2_SO_4_)	77	[139]
Mo_2_C	Precipitation and calcine	Nanoporous	HER	54 (H_2_SO_4_)	200	[140]
α-Mo_2_C	Urea-glass route	Nanoparticles	HER	57 (KOH)	176	[141]
Mo_2_CT_x_	Chemical exfoliation	Nanosheets	HER	189 (H_2_SO_4_)	75	[142]
Mo_2_TiC_2_T_x_	Chemical exfoliation	Nanosheets	HER	248 (H_2_SO_4_)	74	[142]
Mo_2_Ti_2_C_3_T_x_	Chemical exfoliation	Nanosheets	HER	275 (H_2_SO_4_)	99	[142]
TaC NCs@C	Micro-cutting-fragmentation	Nanocrystals	HER	143 (H_2_SO_4_)	146	[143]
Ta-Hf-C	Magnetron sputtering	Films	HER	129 (H_2_SO_4_)	198	[144]
Co_3_W_3_C	TPR	Nanoparticles	OER	59 (KOH)	238	[145]
Ni_0.7_Fe_0.3_PS_3_@MXene	Solid-state reaction	Nanohybrid	OER	36.5(KOH)	282	[146]
Ni-Mo_x_C	Thermal conversion	Graphene/nanotube hybrid	OER	74(KOH)	328	[147]
Fe-Ni_3_C	Carburizing treatment	Nanosheets	OER	62(KOH)	275	[148]
Ti_3_C_2_T_x_ − CoBDC	Interdiffusion reaction	Nanosheets	OER	48.2(KOH)	410	[149]
Co_3_Mo_3_C	TPR	Micrometers particles	HER	93 (KOH)	169	[150]
N-Ti_2_CT_x_	Chemical exfoliation	Nanosheets	HER	67 (H_2_SO_4_)	215	[151]
Co-Mo_2_C	Carbonization	Nanosheets	HER	39 (H_2_SO_4_)	48	[152]
Mo_2_C-C	spray drying and calcination	Flake structure	HER	69 (H_2_SO_4_)	110	[153]
Ti_3_C_2_O_x_	Chemical exfoliation	2D flakes	HER	60.7 (H_2_SO_4_)	190	[154]
W_2_C@GL	Heat treatment	Nanoparticles	HER	68 (H_2_SO_4_)	135	[155]
Mo-WC@NCS	TPR	Nanosheet	HER	81 (KOH)	179	[156]
VC@NC/C	TPR	3D network	HER	165 (KOH)	238	[126]

Metallic alloy effect has also been studied such as Mo-W–C [133–135]. When the Mo/W ratio of this bimetal hollow sphere is adjusted to 1.26/0.74, the overpotentials are 106, 127, and 152 mV corresponding to use 1 M KOH, 0.5 M H_2_SO_4_ and 1 M phosphate buffer as the medium, respectively, indicating its board activity. Specially, the overpotential of the Mo_1.26_W_0.74_C@C in alkaline and acid electrolytes is only 237 and 250 mV at the current density of 300 mA cm^−2^, which is obviously better than most reported electrocatalysts. Chen et al. used a new metal–organic framework derivatization method to synthesize a vertically arranged pure phase porous bimetallic carbide with N-doped carbon as a matrix on a flexible carbon cloth (Co_6_W_6_C@NC/CC). It exhibits excellent OER activity with an overpotential of 286 mV at 10 mA cm^−2^. At the same time, it exhibits an enhanced HER activity with an overpotential of 59 mV at 10 mA cm^−2^. The unique HER activity of bimetallic alloy based TMCs can be mainly attributed to the synergistic effect which not only modulated the electronic structure, activity of the active site, but also tuned its conductivity [134]. Except the Mo- and W-based TMCs, other TMCs have not been well studied. Kou et al. prepared the tantalum carbide nanocrystals (TaC NCs@C) adhered to carbon, which have high refractive index (222) crystal planes. Due to the formation of a transition zone between the carbon layer and the (222) crystal planes of TaC, its stability in the process of preparation and electrochemical reaction is enhanced. TaC nanocrystals have a low overpotential of 146 mV at 10 mA cm^−2^, a large exchange current density of 9.69 × 10^–2^ mA cm^−2^ and excellent cycle stability, which is far superior to other reported group-V metal carbide catalysts [136].

Besides employing as the electrocatalysts, the TMCs have also been used in photocatalysts; however, it is still in infant. Huang et al. used tungsten carbide to degrade organic pollutants by near-infrared photocatalysis. The experimental results are well consisted with the three-dimensional finite element simulation, which prove that plasmon resonance responding from WC nanoparticles can occur on the local surface of the near-infrared light, thereby showing high UV–Visible-NIR full-spectrum absorption and high near-infrared triggered photocurrent response. It has near-infrared photocatalytic degradation performance and the catalytic degradation rate of methylene blue (MB) by WC nanoparticles under near-infrared radiation is up to 50% [137].

### Gas Catalysis and Sensing

**Fig. 10:**
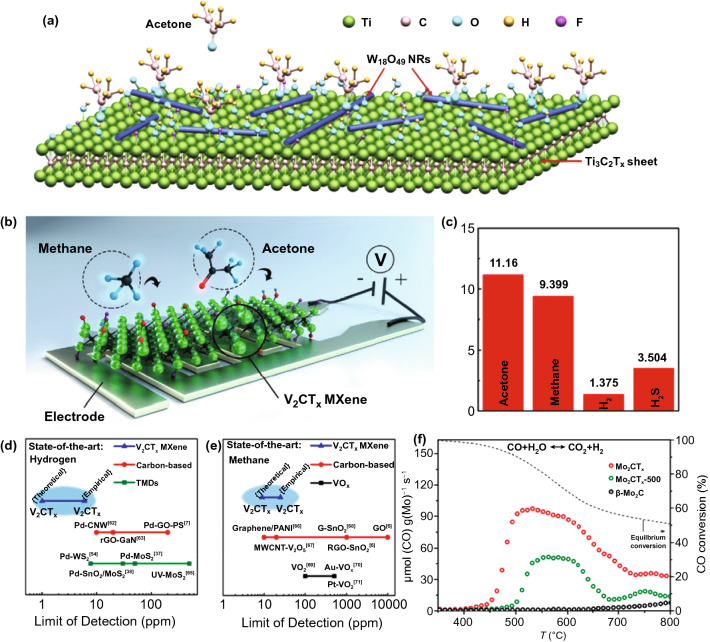
Gas catalytic property of TMCs. **a** Schematic of the reaction between acetone and W_18_O_49_/Ti_3_C_2_T_x_ composite. Reproduced with permission from Ref. [163]. Copyright 2020, Elsevier. **b** Schematic diagram of the sensing mechanism of the V_2_CT_x_ gas sensor. **c** Theoretical LoD of V_2_CT_x_ film toward acetone, methane, hydrogen and hydrogen sulfide at room temperature. Comparison of LoD of **d** hydrogen and **e** methane for room temperature gas sensor. Reproduced with permission from Ref. [161]. Copyright 2019, American Chemical Society. **f** WGS catalytic activity of Mo_2_CT_x_, Mo_2_CT_x_-500, and β-Mo_2_C. Reproduced with permission from Ref. [159]. Copyright 2019, American Chemical Society

The gas molecular conversation would greatly increase the utilization of production in petrochemical industry. The physical and chemical properties of molybdenum carbide with different phases have a significant difference, especially in the field of catalysis. It is known that the catalytic performance of fcc-MoC_1-x_ is different from that of hcp-Mo_2_C in ethane hydrogenation, methanol reforming to hydrogen production, toluene hydrogenation and CO hydrogenation [81]. For example, the CO hydrogenation activity of cubic phase fcc-MoC_1-x_ is twice as high as that of hcp-Mo_2_C hexagonal phase, while hcp-Mo_2_C is more active than fcc-MoC_1-x_ in ethane hydrolysis. In addition, hcp-Mo_2_C nanoribbons with unsaturated Mo sites on surface have higher activity than fcc-Mo_2_C nanoribbons in the dehydrogenation of benzyl alcohol. The different catalytic activities of different phases in molybdenum carbides may be attributed to the influence of surface structure [157]. Dudari et al. prepared molybdenum carbide by Pechini method and CH_4_/H_2_ carburizing gas temperature-programmed reduction method. It was found that the molybdenum carbide prepared by Pechini method mainly contains face-centered cubic MoC_1-x_ phase, while the Mo_2_C phase prepared by TPR method has hexagonal compact packing structure. And the defect phase can be produced by changing the flow rate of Carburizing gas [157]. In addition, the molybdenum carbide has also been demonstrated showing the well catalytic performance in butane dehydrogenation and CO_2_ hydrogenation [158]. Theoretical investigation proves that a rectifying contact is formed at the interface between MoC nanoparticles and nitrogen-doped carbon, which can promote the adsorption and activation of gas molecules, thereby selectively forming formic acid (FA). Molybdenum carbide with different phase structure show different activity and stability for WGS catalytic reaction. The layered Mo_2_CT_x_ had better catalytic activity and stability than other molybdenum carbide structures (as shown in Fig. 7f), and had high selectivity for CO_2_ and H_2_ [159]. The doped molybdenum carbide samples (MoC/N_5.6_C) with significant electron enrichment obtained in the experiment can be used as a stable catalyst to efficiently produce FA through CO_2_ hydrogenation, which is superior to the existing non-precious metals based catalysts. In this study, a durable Schottky heterojunction catalyst with low cost and high performance was designed, which opened up a new way for the application of doped molybdenum carbide in the field of hydrogenation reaction, and further promoted the research on carbon dioxide emission reduction. Except the Mo-based catalysts, Pajares et al. studied the property of VC_x_ with different phases: stoichiometric VC phase and C-deficient V_8_C_7_ phase. On the reverse water gas shift reaction, V_8_C_7_ showed higher CO_2_ conversion rate, CO selectivity, lower apparent activation energy and good chemical stability [160]. Besides as catalysts in gas molecular conversion, the TMCs can also be employed to sense dangerous gas. Sun et al. prepared a composite of one-dimensional Ti_3_C_2_T_x_ and one-dimensional W_18_O_49_ nanorods (as shown in Fig. 10a) [161]. Based on the special interface effect, the composite exhibits high responsiveness to acetone, and has ideal selectivity and long-term stability. Lee prepared layered V_2_CT_x_ MXene on polyimide substrate by chemical exfoliation. The two-dimensional V_2_CT_x_ MXene gas sensor shows ultra-low detection limit (2 ppm) for H_2_ at room temperature, which is better than other two-dimensional gas sensor materials reported at present (as shown in Fig. 10c-e) [161]. In a word, in both catalysis gas conversion and sensing gas, the interface of TMCs and gas molecular plays an important role [162], which could occupy the gas molecular and materials. More efforts should be focused on the interface and improve its occupied mechanism, thus enhancing the interaction.

### Energy Storage

With the environmental crisis, developing energy storage such as battery and supercapacitor has been considered as an environment-friendly strategy. Different with black phosphorene, which has ultra-high diffusivity of Li along the zigzag direction and enhanced electrical conductivity after Li- intercalation [164, 165], TMCs with large specific surface area, good electrical conductivity and excellent cationic intercalation properties have been widely used as electrode materials in energy storage [166]. Nb_2_C and V_2_C show good reversible capacity, high cycle rate and stability, indicating that the rapid diffusion of Li between MXene layers has application prospects in the field of high power [167]. Pang et al. introduced a fluorine-free, simple and rapid method for synthesizing one-dimensional metal carbide nanowires based on three-dimensional Nb_2_CT MXene. The method can synthesize one-dimensional metal carbide nanowires in HCl electrolyte within 4 h. It was found that MXene-based Nb_2_CT nanowires can maintain high stability at a fairly low overpotential (236 mV), and as a water-based zinc-ion battery exhibiting the high power density (420 W kg^−1^) after 150 cycles [77]. V-based TMCs have better performance than many other TMCs and attracted much attention. V_4_C_3_ was used as the anode material of lithium-ion battery, demonstrating that V_4_C_3_ has high capacity, good rate performance and cycle performance. In the case of current density of 0.1 A g^−1^, V_4_C_3_ can still provide a high specific capacity of 225 mAh g^−1^ after 300 charge–discharge cycles [168, 169]. Wang et al. prepared high purity V_2_CT_x_ by a simple hydrothermal assistant method using the mixed solution of NaF and HCl as etchant, and studied the effects of reaction conditions, reaction time and reaction temperature on the reaction yield [137]. It was found that the reaction rate of this system is much faster than that of HF system, and the MAX phase can be etched in three days. At the same time, the electrochemical performance of lithium-ion battery as anode was studied, and it showed a high specific capacitance. When the current was 0.1 A g^−1^, the capacity of lithium-ion battery was 233 mAh g^−1^.

Through chemically etching and exfoliation, the as-prepared Nb_2_CT_x_ nanosheets can provide a high discharge capacity of 354 mAh g^−1^ at a current density of 0.05 A g^−1^. In addition, Nb_2_CT_x_ has good cycle stability, where after 800 cycles at a high current density of 1.0 Ag^−1^, the specific capacity is stable at 225 mAh g^−1^, indicating that Nb_2_CT_x_ can be used as an anode material for LIBs [170]. Nano-NbC decorated N&P-codoped trichoderma spore carbon was synthesized and exhibited an ultra-high rate performance (810 mAhg^−1^ at 5 C) and good cycle stability (937.9 mAh g^−1^ at 0.1 C after 500 cycles) due to the high conductivity attributing to the synergistic effect [171]. Besides, nanocrystalline niobium carbide (NbC) was used as an advanced intermediate layer material for Li–S batteries. The NbC coating combines the anchoring effect of polysulfide (PS) with the advantages of high conductivity, which can effectively inhibit the electrochemical reaction of sulfur and the shuttle of PS. The NbC coating also has excellent cycling stability, the capacity decay rate after 1500 cycles is only 0.037% cycle^−1^, and it has an ultra-high rate capability of up to 5 C, and the area capacity under high sulfur load is as high as 3.6 mAh cm^−2^ [172].

Compared with commercial lithium-ion batteries, rechargeable aluminum batteries have the advantages of safety, cheaper and higher energy density. Table 4 summarizes the recent TMCs-based energy storage development. However, due to the high charge density of Al^3+^ ions and their strong interaction with the host lattice, few Al^3+^ ions can reversibly intercalate these cathode materials. Vahid et al. reported a rechargeable Al-battery based on 2D vanadium carbide (V_2_CT_x_) cathode. The mechanism of charge storage is the reversible intercalation of Al^3+^ ions between V_2_CT_x_ layers. The results show that the electrochemical performance can be significantly improved by converting V_2_CT_x_ particles into multilayer films. The specific capacity of V_2_CT_x_ electrode is more than 300 mAg^−1^_Pt_, and it has higher discharge rate and higher discharge potential, which is one of the best cathode materials for aluminum battery reported at present [173].

**Table 4:** Energy storage performance of transition metal carbides

Sample	Prepare method	Structure	Application	Charge density	Performance	Retention rate	Refs
Ti_3_C_2_T_x_	Chemical exfoliation	Nanosheets	Na-ion battery	0.5 C	103 mAh g^−1^	85.8% after 500 cycles	[181]
Titanium carbide	Chemical exfoliation	Nanorods	Li-ion battery	1 C	843 mAh g^−1^	98.78% after 250 cycles	[182]
Porous- Ti_3_C_2_T_x_	Chemical exfoliation	Nanosheets	Li-ion battery	0.1 C	1250 mAh g^−1^	N/A	[183]
V_2_CT_x_	Chemical exfoliation	Few-layer nanosheets	Al-ion battery	0.5 C	76 mAh g^−1^	96.6% after 100 cycles	[173]
Nb_4_C_3_T_x_	Chemical exfoliation	Layered structure	Li-ion battery	5 C	380 mAh g^−1^	84.2% after 1000 cycles	[184]
Co_3_ZnC	TPR	Microspheres	Li-ion battery	0.5 C	908 mAh g^−1^	67.0% after 300 cycles	[185]
TiO_2_/ Ti_3_C_2_T_x_	Self-assembly	2D heterostructures	Li-ion battery	0.25 C	277 mAh g^−1^	75.5% after 200 cycles	[186]
Nb_2_O_5_@Nb_4_C_3_T_x_	Chemical exfoliation	Layered architecture	Li-ion battery	0.25 C	208 mAh g^−1^	94% after 400 cycles	[187]
Fe_3_C@N–C	Calcinate	Frogspawn-like architecture	Li–S battery	0.5 C	586 mAh g^−1^	99.92% after 400 cycles	[188]
W_2_C NPs-CNFs	TPR	Nanoparticles	Li–S battery	1 C	605 mAh g^−1^	99.4% after 500 cycles	[189]
TiC	Biotemplate method	Nanoflakes	Supercapacitor	5 mV s^−1^	276.1 F g^−1^	94% after 1000 cycles	[190]
Ti_3_C_2_T_x_	Directly annealing	Nanosheets	Supercapacitor	0.5 A g^−1^	442 F g^−1^	95.4% after 5000 cycles	[191]
TaC/C	Laser ablation	Nanospheres	Supercapacitor	1 A g^−1^	223 F g^−1^	94% after 5000 cycles	[192]
MoS_2_/Ti_3_C_2_	Hydrothermal synthesis	2D heterostructures	Supercapacitor	1 A g^−1^	386.7 F g^−1^	91.1% after 20,000 cycles	[193]
MnO_2_-Mo_2_C NFs	Electrospinning	Nanoflakes	Supercapacitor	0.1 A g^−1^	430 F g^−1^	96.1% after 3000 cycles	[194]

Like the MnO_2_, etc., metal oxides which have shown high electrochemical property resulting in high performance electrochemical capacitors [174, 175], the TMCs have also been used as electrode in supercapacitor, which has high power density [176–179]. Xin et al. predicted the application in supercapacitors through ab initio density functional theory considering its quantum capacitance and work function of Nb_n+1_C_n_T_x_. It was found that the niobium carbide with free functional group is suitable for positive electrode, while niobium carbide with functional group has better performance as negative electrode in supercapacitor, showing its broad application prospects in the field of supercapacitor electrode materials. The theoretical quantum capacitances of the positive and negative electrodes are 1828.4 and 1091.1 F g^−1^, respectively [75]. Guan et al. demonstrated that the specific capacitance of chemically exfoliated V_2_CT_x_ MXene can reach up to 164 F g^−1^ and its specific capacitance retention rate can reach 90% after 10,000 cycles at 5 Ag^−1^ [74]. Wang et al. prepared V_2_C layered by carbon nanotubes and studied its electrochemical performance as the electrode of Zn ion supercapacitor, which has a high capacity of 190.2 F g^−1^ at 0.5 Ag^−1^ and excellent cycle stability [139]. Besides, the used electrolyte also significantly affects its supercapacitance; the maximum specific capacitances of V_2_CT_x_ MXene in 1 M H_2_SO_4_, 1 M KOH and 1 M MgSO_4_ solutions are 487, 184 and 225 F g^−1^, respectively, which are the highest among similar micron TMCs electrodes reported [180]. Using seawater as the electrolyte, the supercapacitor based on V_2_CT_x_ MXene has a volume specific capacitance of 317.8 F cm^−3^ at 0.2 A g^−1^ and its capacitance retention rate is 8.1% after 5000 cycles [72]. The supercapacitance of bimetallic TMCs has also been studied. Through studying the influence of ratio of Mo and V in bimetallic MXene, it was found that Mo_2.7_V_1.3_C_3_ has the highest volume capacitance (860 F cm^−3^) and high conductivity (830 S cm^−1^) at room temperature, suggesting that it was able to further optimize the performance by adjusting the element [78].

### Optoelectronic Devices

**Fig. 11:**
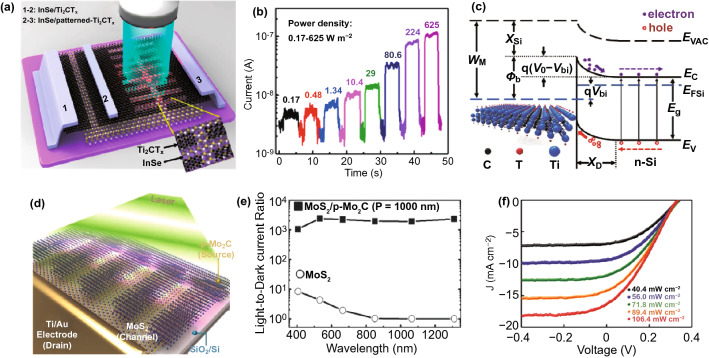
Photoelectrical property of TMCs. **a** Schematic of unpatterned and patterned InSe/Ti_2_CT_x_photodetectors. **b** Photoresponse curves of patterned InSe/Ti_2_CT_x_ avalanche photodetector under different illumination densities. Reproduced with permission from Ref. [197]. Copyright 2019, American Chemical Society. **c** Energy band diagram of Ti_3_C_2_T_x_/n-Si Schottky junction upon illumination [199]. **d** Schematic figure of photodetector device with MoS_2_/p-Mo_2_C hybrid structure under illumination. **e** I_D_–V_G_ curves of photodetector device with MoS_2_/p-Mo_2_C hybrid structure under illumination with various wavelengths. Reproduced with permission from Ref. [201]. Copyright 2019, WILEY–VCH. **f** J–V curves of Ti_3_C_2_T_x_/n-Si heterostructure device under various energy density illuminations. Reproduced with permission from Ref. [199]. Copyright 2017, WILEY–VCH

The unique electronic structure and properties of TMCs, such as the high carrier concentration and high transmittance, would lead an enhanced interaction between phonon and matter, resulting in high photoresponse. Comparatively, the Ti-based MTCs photodetectors have got more investigated not only being employed as transparent electrode, but also being employed as reasonable materials in junction-based photodetector. Significantly, due to the tunable work function by modulating its surface terminated group, the efficiency of junction-based photodetector could be easily improved. It was found that the MXene coated leaf vein network has a high transmittance (about 90%) and low square resistance (3 Ω sq^−1^). The results show that the work function of the MXene electrode can be adjusted by changing the terminal atoms. The MXene electrode and electrospun TiO_2_ film were integrated to construct a translucent UV photodetector, which has high UV detection performance, excellent flexibility and stability, and can withstand 1000 bending cycles [195, 196]. Yang et al. fabricated an InSe photodetector using Ti_2_CT_x_ as the electrodes, as shown in Fig. 11a, b. Because the Ti_2_CT_x_ electrode produces avalanche carrier multiplication effect, the photodetector has excellent photoelectric performance. In addition, the pattern of the Ti_2_CT_x_ electrode into a plasmonic grating structure can further enhance the light absorption, achieving a dark current as low as 3 nA, a responsivity as high as 1 × 10^5^ A W^−1^, a high detection rate (7.3 × 10^12^ Jones) and a shorter light response time (0.5 ms) [197].

Combining 2D Ti_3_C_2_T_x_ with perovskite through top-down technology, it was able to design a large-scale image sensor array consisting of 25 groups of 50 pixels. Due to the good work function matching between the Ti_3_C_2_T_x_ layer and the perovskite active layer, it is helpful to form an effective interfacial charge transfer. The energy level alignment and resonance enhancement of the composite system can optimize the near-infrared absorption of the composite system. The results show that the device has excellent broadband spectral response, a response rate of 84.77 A W^−1^, a specific detection rate of 3.22 × 10^12^ Jones, a linear dynamic range up to 82 dB and a near-infrared image capture capability [198]. Kang prepared the vertically Ti_3_C_2_T_x_/n-Si Schottky heterojunction (as shown in Fig. 11c, f) and demonstrated that it has an open-circuit voltage of 0.34 V and a short-circuit current density of 12.9 mA cm^−2^ under 100 mW cm^−2^ illumination, an I_ph_/I_dark_ ratio of about 10^5^, a responsivity of 26.95 mA W^−1^, a response time of 0.84 ms and a recovery time of 1.67 ms [199].

Other TMCs-based photoelectronic effects have also been studied. It is found that the relaxation time of Nb_2_C nanosheets can be tunable in the range from 37.43 fs to 0.57 ps by optimizing its size. The layered Nb_2_C nanosheets have promising potential applications in broadband ultrafast photonics and near-infrared photonic devices [200]. Jeon et al. demonstrated that the chemical vapor deposition grown MoS_2_ film can be chemical converted to Mo_2_C film, as shown in Fig. 11d, e. Using the interface characteristics of MoS_2_ and Mo_2_C, that is, effective hot carrier injection from Mo_2_C to MoS_2_, the photodetector has high sensitivity and spectral response performance. By adjusting the grating period (400 ~ 1000 nm) of Mo_2_C, a broad-spectrum response of light (655 ~ 1200 nm) can be achieved. The results show that the photodetector has high responsivity (R > 10^3^ A W^−1^) and bright-dark current ratio (> 10^2^) in a wide spectral range (405 ~ 1310 nm), which is similar with that of transition metal dichalcogenides [201–203].

### Medical Treatment

**Fig. 12:**
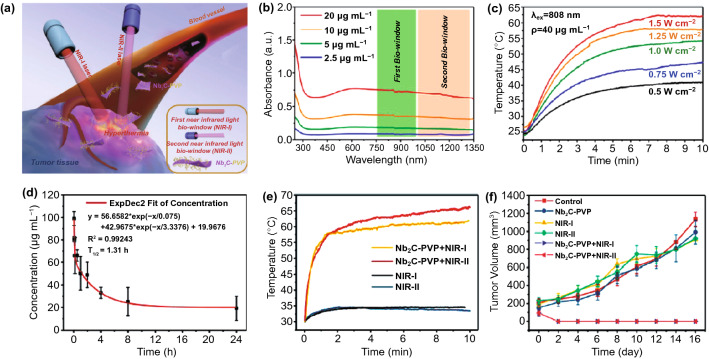
Medical treatment based on Nb_2_C. **a** Schematic diagram of two-dimensional Nb_2_C used in photothermal treatment of tumors. **b** S-NIR absorption spectra of different concentrations of Nb_2_C NSs aqueous suspension. **c** Photothermal curve of Nb_2_C NSs aqueous suspension irradiated by 808 nm near-infrared light irradiation at different power densities. **d** Blood circulation lifetime of Nb_2_C − PVP after intravenous injection into mice. **e** Temperature elevations at the tumor sites of 4T1-tumor-bearing mice in groups of NIR-I, NIR-II, Nb_2_C − PVP + NIR-I and Nb_2_C − PVP + NIR-II during laser irradiation. **f** Time-dependent tumor growth curves after different treatments (control, Nb_2_C − PVP only, NIR-I, NIR-II, Nb_2_C − PVP + NIR-I and Nb_2_C − PVP + NIR-II). Reproduced with permission from Ref. [204]. Copyright 2017, American Chemical Society

Due to the well photoelectronic and photothermal property, the TMCs have also been well used in medical treatment. Jastrzebska et al. proved for the first time that the highly negative surface charge of niobium carbide can be basically transformed into a high positive charge by surface modification with poly L-lysine (PLL). The conversion of surface charge will enable niobium carbide to obtain important biological effects, such as targeting tumors and inducing programmed cell death in G0/G1 phase, which are the most ideal effects for the design of tumor targeting drugs. Significantly, the biocompatibility of PLL modified niobium carbide (Nb_2_C and Nb_4_C_3_) is better than that of unmodified niobium carbide [51]. Furthermore, Nb_2_C modified by PVP has been proofed effectively eliminate mouse tumor xenografts in NIR-I and NIR-II bio-windows, as shown in Fig. 12. Two-dimensional Nb_2_C nanosheets have excellent photothermal conversion efficiency (36.4% for NIR-I, 45.65% for NIR-II), and good photothermal stability. In addition, Nb_2_C nanosheets also have unique enzyme-responsive biodegradability to human myeloperoxidase [204]. 2D Nb_2_C nanosheets have been demonstrated with excellent antioxidant properties and can effectively scavenge hydrogen peroxide, hydroxyl radicals and superoxide radicals. The polyvinylpyrrolidone (PVP) modifying would significantly improve its biocompatibility, resulting in an effective protective effect on the hematopoietic system, testis, small intestine and lung of γ-ray irradiated mice, in particular, the hematopoietic system. Experiments show that Nb_2_C-PVP can be effectively eliminated by the liver and kidneys in mice after 14 days [205].

V_2_C, as a photothermal agent with excellent photothermal conversion efficiency, has a great application prospects in the field of photothermal therapy. Zada et al. reported a new exfoliation method, that is, the parent phase MAX is intercalated and delaminated by algae extract, and V_2_C nanowires with complete structure and high NIR absorption capacity are obtained. Through characterization, the photothermal conversion efficiency of the prepared V_2_C nanosheets is as high as 48% [50]. Lin et al. used HF as an etchant to synthesize two-dimensional Ta_4_C_3_ by chemical exfoliation, and explored its application in the photothermal ablation of tumors in vivo. It was proved that the soybean phospholipid-modified Ta_4_C_3_ has good biocompatibility, excellent performance of photothermal conversion (efficiency η of 44.7%) and in vitro/in vivo photothermal ablation of tumors [206]. The ionizing radiation generated by radiation accident will has a serious impact on exposed individuals. In addition, by functionalizing with MnO_x_, the Ta_4_C_3_ is expected to be widely used in the field of tumor synergistic therapy based on its photothermal conversion performance, tumor microenvironment (TME)-responsive T_1_-weighted MR imaging capability and as the desirable contrast agents for PA imaging [35].

### Superconductor

**Fig. 13:**
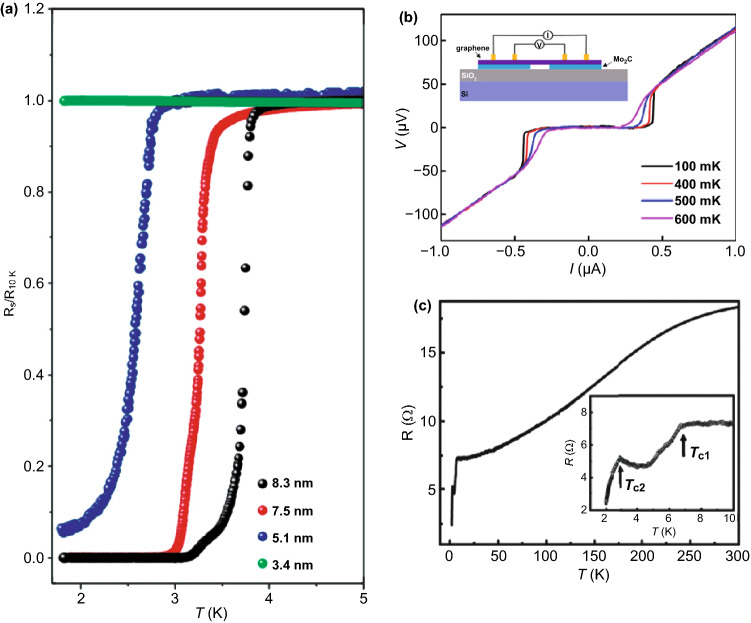
Superconductivity characteristic of metal carbides. **a** Superconductivity characteristic of Mo_2_C flakes with different thickness. Reproduced with permission from Ref. [90]. Copyright 2015, Springer Nature. **b** Typical dc Josephson response of Mo_2_C/graphene/Mo_2_C junction taken at different temperatures. Reproduced with permission from Ref. [95]. Copyright 2017, American Chemical Society. **c** Superconductivity characteristic of NbC flakes. Reproduced with permission from Ref. [211]. Copyright 2020, American Physical Society

Due to the high carrier concentration and strong correlation system, the TMCs have been proved as a new superconductor member obtaining great attention. Using CVD-grown Mo_2_C as the model, Xu et al. demonstrated that the superconducting properties are consistent with the Berezinskii–Kosterlitz–Thouless behavior, and the superconducting properties depend on the crystal thickness, as shown in Fig. 13. Significantly, 2D Mo_2_C crystals also show strong magnetic anisotropy [90]. Furthermore, the graphene/2D α-Mo_2_C structure has a superconducting transition phase diagram with multiple voltage steps in the transition zone, which is expected to be widely used in the field of highly transparent Josephson junction devices [95]. In addition, the influence of grain boundaries on the electron transport and superconductivity properties of 2D Mo_2_C were explored. In the normal state, with the increase in grain boundary inclination angle, the critical current decreases by 1 to 2 orders of magnitude during the transition from superconducting state to resistive state. In the superconducting state, crossing the grain boundary will lead to the critical current to decrease significantly [207]. Jin et al. prepared double-layer Mo_2_Ga_2_C by vacuum hot pressing. It was found that the RT thermal conductivity of Mo_2_Ga_2_C is 14.8 ± 1.0 W (m K)^−1^, the RT resistivity is 0.525 ± 0.052 μΩ m, and the Lorenz number is 2.22 × 10^−8^ WΩ K^−2^. Importantly, Mo_2_Ga_2_C has superconductivity, and the superconducting transition temperature is 5.1 K [208]. Porrati used Nb(NMe_2_)_3_(N-t-Bu) as the precursor to prepare two-dimensional nanowires and self-supporting three-dimensional nanowires through focused electron beam-induced deposition (FEBID) and focused ion beam-induced deposition (FIBID). Electrical transmission measurements show that FEBID nanowires are insulated, FIBID two-dimensional nanowires have superconductivity at T_c_≈5 K, and the critical superconducting temperature of self-supporting FIBID three-dimensional nanowires reaches T_c_≈11 K. The results show that FIBID-NbC has broad application prospects in the preparation of superconducting nanowire single-photon detectors and quantum information processing, suggesting that the property is dependent on its dimensionality [209, 210].

### Other Potential Applications

Similar with other 2D materials, such as graphene, phosphorene and transition metal dichalcogenides (e.g., MoS_2_ and WS_2_), which has shown great potential for thermal management and thermoelectric energy generation [212, 213], the thermal and thermoelectric properties of the TMCs, especially the MXene (Ti_3_C_2_T_x_), have been studied. Due to its high electrical conductivity, TMCs have been usually composited in thermoelectric materials-based matrix. It was reported that compositing MXene (Ti_3_C_2_T_x_) into (Bi,Sb)_2_Te_3_ matrix can simultaneously improve power factor and reduce thermal conductivity. Under a temperature gradient of 237 K, the thermoelectric conversion efficiency reached a record of 7.8% [214]. Hong et al. reported the sub-nano ion channel based on 2D-TMCs, which can convert external temperature changes into electrical signals through the preferential diffusion of cations under a thermal gradient. Based on the photothermal conversion of MXenes, the Ti_3_C_2_T_x_ ion channel can capture the diffusion potential across the nanochannel under the axial temperature gradient of the light drive and exhibit the photothermoelectric ionic response of 1 mV K^–1^ [215].

Recently, TMCs have also been used to fabricate nanofiltration membranes due to its uniform nanopores. The TMCs-based nanofiltration membranes show extraordinary molecular separation performance. Kim et al. reported a slot-die coating method to prepare large-area Ti_3_C_2_T_x_ MXene. The Ti_3_C_2_T_x_ membrane exhibits excellent nanofiltration performance, which deliver a water permeability of 190 LMH/bar and a molecular weight cutoff rate of 269 Da [216]. Stable interlayer space is the key factor to improve ion selectivity. Wang et al. proposed a strategy to stabilize Ti_3_C_2_T_x_ layered structure through alginate hydrogel pillars. The membrane has good permeation cutoff and screening performance for valence cations. Moreover, its excellent H^+^/Fe^2+^ selectivity makes this membrane promising as an ion-exchange membrane. Recently, the Ti_3_C_2_T_x_ stabilized by the alginate hydrogel pillars with the same d-spacing has 100% Na_2_SO_4_ rejection and high water permeability [217]. Xu et al. developed a new way to use MXene nanosheets to overcome the trade-off limitation of membrane permeability and salt selectivity. The thin-film composite nanofiltration membranes have a permeability of 45.7 L m ^−2^ h ^−1^ bar ^−1^, and a Na_2_SO_4_ removal rate of 96% [218].

Due to its high electrical conductivity and high light adsorption, TMCs have also been used in electromagnetic shielding. Rajavel et al. prepared a flexible few-layer Ti_3_C_2_T_x_ film. At a thickness of 6 μm, the X-band conductivity is about 3669 ± 33 S m^−1^, and the electromagnetic interference shielding efficiency is 31.97 dB. It has also been demonstrated that through controlling the inherent defects, it was able to adjust the electromagnetic shielding performance of few-layer Ti_3_C_2_T_x_ [219]. Aïssa et al. prepared a two-dimensional Ti_3_C_2_T_x_ MXene/GNPs composite film using electrohydrodynamic atomization deposition technique. The Ti_3_C_2_T_x_ MXene/GNP film with the thickness of 1.75 mm shows excellent electromagnetic shielding performance, with an electromagnetic interference absorbance of about 64 dB [220]. Further optimizing the fabrication of TMCs and designing the device could improve its performance.

Hydrogen energy is one of the most promising clean energy sources. However, the current hydrogen storage materials still not very well satisfy the industrial requirement. Due to the higher surface activity and larger surface area, recently, the hydrogen storage performance of the incomplete etched Ti_2_CT_x_ MXene film has been investigated, and the hydrogen storage mechanism has been discussed [221]. It was found that the Ti_2_CT_x_ film has excellent hydrogen storage efficiency, where 8.8 wt% hydrogen is completely absorbed at room temperature under the environment of 60 bar H_2_. The small interlayer distance of Ti_2_CT_x_ MXene and the F functional group brought by etching are the key to its hydrogen storage, which will induce weak chemical adsorption assisted by nano-effects. Noh et al. used a two-step method to synthesize palladium nanoparticle-decorated multilayer Ti_3_C_2_T_x_ MXene (Pd-Ti_3_C_2_T_x_) [222]. It was found that Pd-Ti_3_C_2_T_x_ exhibits typical hydrogen storage capacity at room temperature and 77 K. Zhu et al. self-assembled Ni nanoparticles on Ti_3_C_2_ MXene obtained by etching and then composited with MgH_2_ by ball milling. The MgH_2_ + Ni@Ti-MX composite material can absorb 5.4 wt% H_2_ at 125 ℃ for 25 s, and release 5.2 wt% H_2_ at 250 ℃ for 15 min [223]. Although the TMCs materials already exhibit the potential application in the hydrogen storage, further study including the correlation between the materials structure and its performance is still unclear and more efforts still need.

## Conclusions and Perspectives

This article reviews the progress in the structure, properties, applications and synthesis methods of transition metal carbides represented by niobium carbide, vanadium carbide, molybdenum carbide and titanium carbide. Firstly, the different phase structures of four typical transition metal carbides are introduced. Different TMCs have the basic phase of NaCl-type cubic phase, and all group IV and V TCMs have M_6_C_5_ phase. Under different synthesis processes, carbon atoms and vacancies are rearranged to varying degrees, resulting in a variety of stable phase structures of transition metal carbides. Because the existence of different phase compositions gives transition metal carbides rich and diverse properties, they have been researched and developed in different fields. Based on their outstanding electronic, mechanical, magnetic, electrochemical, optical properties and atomic-level thickness, TMCs films have been applied in the fields of catalysis, energy storage, optoelectronics, biomedicine and superconductivity. The hydrogen adsorption on the surface of transition metal end carbides is significantly enhanced, which makes it good catalyst for electrocatalytic and photocatalytic hydrogen evolution. Due to the large specific surface area, good conductivity and excellent cation intercalation performance, TMCs have potential application in LIBs anode materials. Due to the rectifying electric shock with Schottky barrier height and internal electric field, and higher responsivity and quantum efficiency than Au, Ti_3_C_2_T_x_ photodetector has better dynamic range and detection rate. The surface charge of Nb_2_C and Nb_4_C_3_ is transformed into high positive charge by surface modification, which has important biological effect on tumor targeting. TMC films such as α-Mo_2_C and NbC have superconducting properties, and the critical superconducting temperature can be further increased by changing the synthesis process and adjusting the surface functional groups.

At present, the main ways to synthesize TMCs films include chemical exfoliation, chemical vapor deposition, temperature-programmed reduction and magnetic sputtering. In recent years, MXenes prepared by chemical exfoliation have been developed rapidly, and its layer structure similar to accordion has brought a wealth of applications. The TMCs synthesized by chemical vapor deposition have high purity and are easy to be uniformly doped. Temperature-programmed reduction provides a way for the synthesis of carbides with high synthesis temperature. Magnetic sputtering synthetic carbide film has the characteristics of high film formation rate, low substrate temperature and good film adhesion.

Although TMCs films have shown great potential in different fields, there are still some challenges in future applications. First of all, the current synthesis methods of TMC films still face some limitations. For example, it is difficult to synthesize the parent phase MAX of MXene, and many MXenes have not been successfully prepared by chemical exfoliation because of the inability to synthesize the stable MAX phase. Chemical vapor deposition has limitations in the preparation of large-size carbide films. Therefore, new methods for synthesizing TMCs have yet to be explored. Secondly, the theoretical mechanism of some characteristics of TMC films in the application field is still unclear. For example, in the field of energy storage, the ion dynamics and charge storage mechanism between carbide films are unclear. Thirdly, the improvement of electrochemical, mechanical and thermal stability of TMCs remains a topic of future research. It is worth mentioning that the research fields of TMCs films are full of opportunities and challenges, and there is still great application potential to be tapped in different fields. In the foreseeable future, transition metal carbide materials will play an increasingly important role in solving various global challenges.
